# 

**Published:** 1871-05

**Authors:** 


### INDEX OF SUBJECTS.

### ORIGINAL COMMUNICATIONS.

Gynæcological Medicine.......218
Hypodermic Medication.......227
Transactions of the Macon Medical Association.......235

### EXTRACTS AND GLEANINGS.

Tubal Pregnancy, 237; Carbuncle—The Incubation Period of Syphilis, 238; Belladonna in Typhoid Fever—Dropsical Effusions, 239; Cauterization in Diptheria—Improved formula for Chalk Mixture, 240; Mur. Ammonia in Nervous affections—Lime water in Bright's Disease, 241; Cough Mixtures, 242; Contraction of limb cured by subcutaneous injection of Atrophia—Rapid cure for Buboes, 243; Turpentine in Uterine Hemorrhage—Ergotine after Amputation, 244; Treatment of Herpes Zoster by Belladonna Ointment—Sulphurious acid in Typhoid Fever, 245; Calabar Bean in Tetanus, etc.—Cardiac Neuralgia—Application for Cancer, 246; Things not generally known—Gleet and Leucorrhœa cured by Bromide Potassium—Possible duration of Pregnancy, 247; Speaking and Singing without a Tongue, 248; Epistaxis—Therapeutic effects of Gelseminum, 249; Purpura in Children—Treatment of Itch, etc., 250; Camphor as an application in chancre—Chloral Hydrate in Convulsions, 251; Value of Conium—A new Tonic—Remedy for Diptheria, 252; Remedy for Asthma, 253; A new Anæsthetic—Oil Peppermint an Anæsthetic, 254; Carbolic Acid in Pruritus—Treatment of Bursæ—Treatment of Orchitis, 255; Incontinence of Urine—Nitric Acid in Bright's Disease—Skin Affections etc. 256; Abscess of the Mastoid cells cured, etc.—Castration for Epileptic Insanity, 257.

### CORRESPONDENCE—HYGENIC AND MISCELLANEOUS.

Our Carolina Correspondent.......258
Letter from a distinguished Medical Gentleman.......259
Influence of Athletic Sports.......261
Power of Nature in the cure of Disease—Dyspepsia treated with Raw Meat.263

### EDITORIALS, REVIEWS, ETC.

A Victory.......265
The Georgia Medical Association.......268
Medical Association of Macon.......269
Letter from a Distinguished Medical Gentleman.......270
Macon Medical Association.......271
An Interesting Letter.......273
New Hypodermic Syringe.......274

